# Research frontiers and trends in the application of artificial intelligence to sepsis: A bibliometric analysis

**DOI:** 10.3389/fmed.2022.1043589

**Published:** 2023-01-12

**Authors:** Meng Tang, Fei Mu, Chen Cui, Jin-Yi Zhao, Rui Lin, Ke-xin Sun, Yue Guan, Jing-Wen Wang

**Affiliations:** Department of Pharmacy, Xijing Hospital, Fourth Military Medical University, Xi’an, China

**Keywords:** artificial intelligence, sepsis, bibliometric analysis, CiteSpace, VOSviewer

## Abstract

**Background:**

With the increasing interest of academics in the application of artificial intelligence to sepsis, thousands of papers on this field had been published in the past few decades. It is difficult for researchers to understand the themes and latest research frontiers in this field from a multi-dimensional perspective. Consequently, the purpose of this study is to analyze the relevant literature in the application of artificial intelligence to sepsis through bibliometrics software, so as to better understand the development status, study the core hotspots and future development trends of this field.

**Methods:**

We collected relevant publications in the application of artificial intelligence to sepsis from the Web of Science Core Collection in 2000 to 2021. The type of publication was limited to articles and reviews, and language was limited to English. Research cooperation network, journals, cited references, keywords in this field were visually analyzed by using CiteSpace, VOSviewer, and COOC software.

**Results:**

A total of 8,481 publications in the application of artificial intelligence to sepsis between 2000 and 2021 were included, involving 8,132 articles and 349 reviews. Over the past 22 years, the annual number of publications had gradually increased exponentially. The USA was the most productive country, followed by China. Harvard University, Schuetz, Philipp, and *Intensive Care Medicine* were the most productive institution, author, and journal, respectively. Vincent, Jl and *Critical Care Medicine* were the most cited author and cited journal, respectively. Several conclusions can be drawn from the analysis of the cited references, including the following: screening and identification of sepsis biomarkers, treatment and related complications of sepsis, and precise treatment of sepsis. Moreover, there were a spike in searches relating to machine learning, antibiotic resistance and accuracy based on burst detection analysis.

**Conclusion:**

This study conducted a comprehensive and objective analysis of the publications on the application of artificial intelligence in sepsis. It can be predicted that precise treatment of sepsis through machine learning technology is still research hotspot in this field.

## 1. Introduction

Sepsis, a life-threatening organ dysfunction caused by a dysregulated host response to infection, is one of the severe challenges facing global public health (1). It is known that sepsis has a high mortality rate as well as a high morbidity rate. A meta-analysis showed that more than 19 million people had p sepsis in worldwide each year, including at least 5 million deaths (2). A study in China found an annual standardized sepsis-related mortality rate of 66.7 per 100,000 people, with an estimated that more than 1 million people deaths from sepsis nationwide (3). There was a evidence that early identification and diagnosis were critical, and appropriate interventions can significantly improve the prognosis of patients with sepsis (4, 5). However, due to the complex pathophysiological conditions of sepsis, individual differences, and delayed laboratory results, it often leads to the lack of early detection and treatment of sepsis (6, 7).

A great deal of progress has been made in the field of artificial intelligence, which utilizes machine algorithms to simulate human cognitive functions, such as graphics and sounds recognition, learning, reasoning, generalization, and problem-solving, which made it widely used in related fields such as disease diagnosis, medical imaging, personalized treatment (8–11). This has contributed to the development of precision medicine.

At present, machine learning methods based on artificial intelligence are widely used in early diagnosis, individualized treatment, and disease stratification of sepsis to improve clinical practice and patient prognosis. It is well known that the results of blood culture are the gold standard for the diagnosis of sepsis, however because of the slow and complicated culture results, the treatment of patients are often delayed. The application of artificial intelligence technology has greatly improved the diagnosis speed of sepsis. For example, Henry KE et al. used conventional physiological and laboratory data to develop a real-time warning score to predict which patients will develop septic shock and reduce the mortality of patients with sepsis (12). Joon-myoung et al. developed a model based on deep learning to screen sepsis using electrocardiogram (13). With the increasing interest of researchers in the application of artificial intelligence in sepsis, thousands of papers on this field have been published in the past 22 years. It is difficult for researchers to understand the themes and latest research hotspots in this field from a multi-dimensional perspective.

Bibliometrics takes publications as the research object, and uses statistics, mathematics and other measurement methods to present the overall development, thematic research, research hotspots and other issues in a certain field (14, 15). Bibliometrics tools have powerful analysis and visualization capabilities. They can not only analyze thousands or even tens of thousands of documents, but also analyze and visualize scientific research cooperation from macro and micro perspectives. The biggest feature is that they can analyze and visualize research hotspots in specific fields from different dimensions. These advantages are not available in traditional literature review and meta-analysis. In recent years, bibliometrics has been widely used to analyze different fields such as prostate cancer, flash translation layer, health education economic and Apache Hadoop etc. (16–19). However, there is no publication using bibliometrics to analyze application of artificial intelligence to sepsis.

We collected relevant publication on the application of artificial intelligence to sepsis from the WOSCC database between 2000 to 2021, and used bibliometric analysis software to analyze countries, institutions, authors, journals, cited journals, cited references and keywords. The main purpose is to understand the overall development of this field, the core strength of development, the hotspots and trends of research topics. It is hope that it can provide direction for researchers interested in this field.

## 2. Materials and methods

### 2.1. Data sources and searching strategy

**FIGURE 1:**
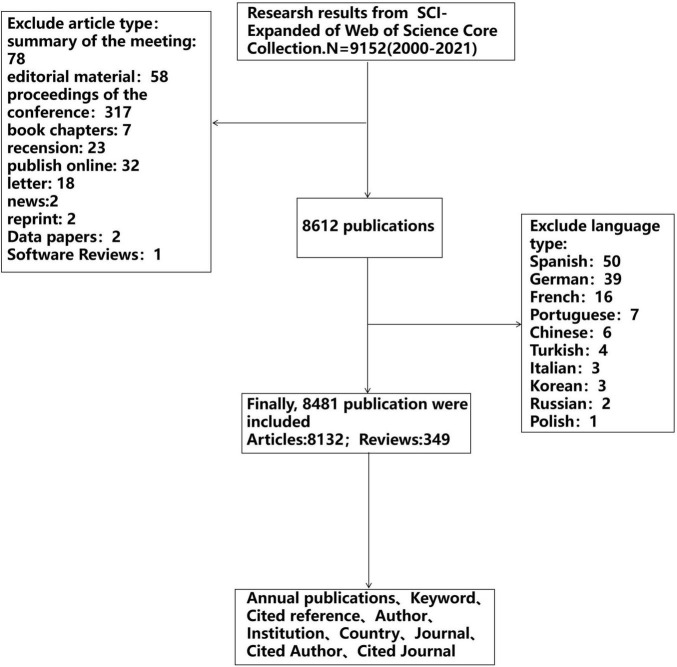
Flowchart of the retrieval strategy in the study.

We used Science Citation Index Expanded (SCIE) of Web of Science Core Collection (WOSCC) to retrieve relevant publications. As we all know, the database has a relatively reliable database covering more than 12,000 of the most influential high-quality scientific journals and is widely used for scientometric analysis (20–22). Our search terms combine Medical Subject Headings and keywords, such as sepsis, machine learning. The complete search strategy was shown in Supplementary material. To avoid data updates, we retrieved the data within a day. The search years were 2000–2021, the publications type was limited to article and review, and the language was limited to English, as shown in Figure 1. The downloaded data included titles, authors, year of publication, countries/regions, institutions, keywords, abstracts, references, etc. The document was downloaded in plain text format and tabular separator.

### 2.2. Data analysis and visualization

The data were mainly analyzed by Microsoft excel 2019, CiteSpace, VOSviewer, and COOC.

Microsoft Excel 2019 was designed to analyze and draw the number and trend of publications published each year, the number of journal publications and their impact factors, and the relevant information of cited references, etc.

Our data was preprocessed before analysis, and the specific steps were as follows: (1) Firstly, we imported the data into CiteSpace for deduplication (23); (2) Secondly, we converted the format of the data in CiteSpace (24); (3) Finally, we cleaned the data. In the country analysis, Wales, Scotland, North Ireland, and England were classified as the UK, and Taiwan was classified as China (25). In terms of institutions, for the organization of writing unification, such as, Univ Texas Southwestern med CTR Dalla was classified as Univ Texas SW med CTR Dallas; In keywords analysis, synonymous keywords were merged, (26) such as intensive care unit (ICU)was classified intensive care unit, acute kidney injury (AKI) was classified acute kidney injury. Complete data cleansing can be found in Supplementary material.

CiteSpace is an information visualization software based on Java language, which presents the rules, structure and distribution of scientific knowledge (27). Through the analysis of the cited references, CiteSpace can reveal the knowledge structure of a certain research field, the evolution of the research frontier, and the publications that plays a key role in the evolution process. Through the analysis of keyword burst, the keywords with high active degree in a certain period can be extracted to find the decline or rise of keywords (24). Therefore, CiteSpace was used for clustering and time line view analysis of cited references and analysis burst of keywords. The parameters of cited references were selected as follows: Time span: 2000–2021; slice year: 2; Threshold value: g-index (*K* = 25) LRF = 3, L/N = 10, *E* = 1.0, No pruning mode. The following parameters were used to analyze the prominence of keywords: time span: 2000–2021, slice year: 2; Select criteria Top 10%, No pruning mode.

VOSviewer is a software developed based on Java language for building and visualizing bibliometric networks. It can display the structure, evolution and cooperation of knowledge fields. The outstanding feature of VOSviewer is that it has strong graphic display ability and is suitable for large-scale data analysis (28). In this paper, VOSviewer was used to analyze the research collaboration network, excavated the relationship between countries/regions, institutions and authors, and conducted cluster analysis. Parameters were as follows: Country: minimum number of citations of a country: 0, minimum number of documents of a country: 5; Institutions: minimum number of citations of an organization: 0; minimum number of documents of an organization: 10; Author: minimum number of citations of an author: 0, minimum numbers of documents of an author: 6; Cited Author: minimum number of citations of an author: 50; Keywords: minimum number of occurrences of a keyword: 8.

COOC is a multi-functional bibliometric software, which can be used to analyze synonyms, frequency statistics, co-occurrence matrix, dissimilarity matrix and word matrix (29). This paper used COOC software to visually analyze keywords, journals and cited journals. Parameter: Top 10 keywords, journals and cited journals were selected for analysis.

## 3. Results

### 3.1. Annual trends in publications and citations

**FIGURE 2:**
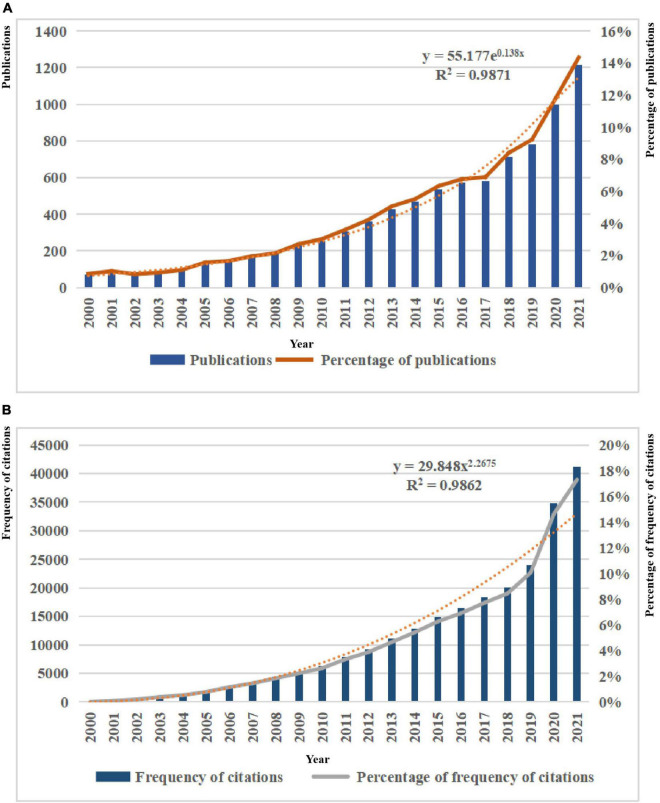
**(A)** Trends in the number of publications on the application of artificial intelligence in sepsis from 2000 to 2021. **(B)** Trends in the number of frequency of citations on the application of artificial intelligence in sepsis from 2000 to 2021.

A total of 8,481 publications on the application of artificial intelligence to sepsis published between 2000 and 2021 were included, including 8,132 articles and 349 reviews. Over the past 22 years, there had been a gradual increase in the number of publications per year, from 73 (1%) originally published in 2000 to 1,217 (14%) published in 2021. By fitting the data, we observed that the number of publications increases exponentially with year (*R*^2^ = 0.9871), as shown in Figure 2A. In addition, the citation frequency of publications increased from 15 (0.01%) in 2000 to 41,151 (17%) in 2021, and the fitting data showed that the citation frequency increased with the power of the year (*R*^2^ = 0.9862), as shown in Figure 2B.

### 3.2. Analysis of top productive countries/regions and institutions

**FIGURE 3:**
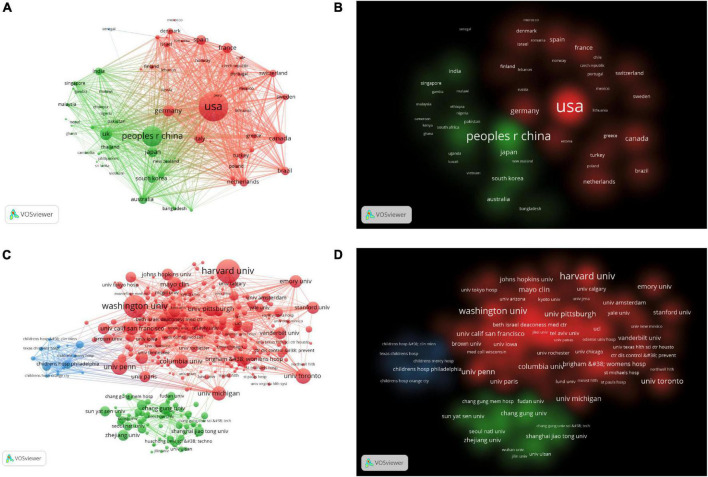
**(A)** The citation network visualization map of countries/regions. **(B)** The citation density visualization map of countries/regions. **(C)** The citation network visualization map of institutions. **(D)** The citation density visualization map of institutions.

There were 79 countries that had published more than five articles in this field. Table 1 showed that the top 10 countries/regions and institutions in the number of published articles. The top three countries/regions were USA (*n* = 893, 36.74%), China (*n* = 392, 17.97%), and UK (*n* = 157, 6%). As shown in Figure 3A, we used VOSviewer to visualize the cooperation relationship between various countries. The most obvious was that the USA had cooperated with many countries, such as France, China, Germany, etc. The lines represent cooperation between countries, and the more cooperation, the thicker the lines. The top five countries for total link strength were USA, China, UK, Canada, and Germany. Figure 3B was the visualization of cluster density. The red cluster showed that there were many projects around the USA, which were accounting for a large weight, while the green cluster showed that there were many projects around China, accounting for a large proportion.

**TABLE 1:** The top 10 countries/regions and institutions with most publications on the application of artificial intelligence in sepsis from 2000 to 2021.

Rank	Country/ Regions	Count	Total link strength	Rank	Institution	Count	Total link strength	Country
1	USA	3116	1750	1	Harvard University	224	482	USA
2	China	1524	576	2	Washington University	206	435	USA
3	UK	546	875	3	University of Pennsylvania	128	257	USA
4	Canada	483	703	4	University of Toronto	119	383	Canada
5	Germany	472	738	5	University of Pittsburgh	117	287	USA
6	Japan	381	226	6	Columbia University	108	280	USA
7	France	363	559	7	Mayo Clinic	106	187	USA
8	Spain	358	556	8	University of Michigan	105	320	USA
9	Italy	328	657	9	University of California San Francisco	92	227	USA
10	Australia	294	577	10	Emory University	90	259	USA

There were 452 organizations that published more than 10 articles, as shown in Figures 3C, D. The top 10 institutions contributed 1,295 articles, accounting for 15.27% of all publications. The top three institutions Harvard University (*n* = 224), Washington University, (*n* = 206), University of Pennsylvania (*n* = 128) from the USA. As shown in Table 1, nine of the top 10 institutions were from the USA and one was from Canada. There was extensive cooperation between most institutions. The top three institutions for TLS were Harvard University, Washington University, University of Toronto.

### 3.3. Analysis of authors and co-cited authors

**FIGURE 4:**
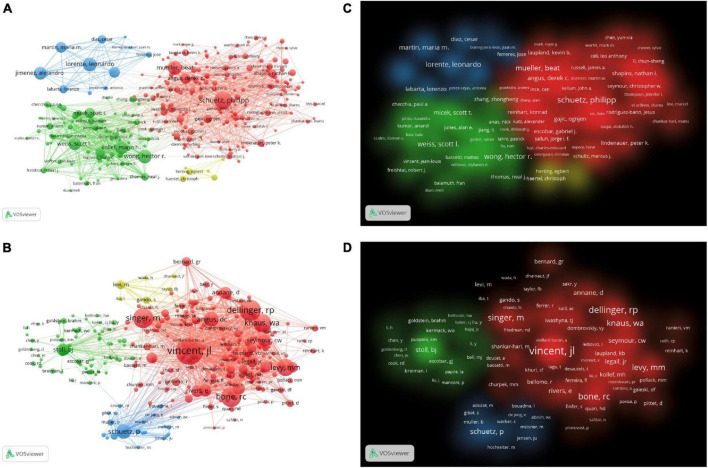
**(A)** The network visualization map of co-authorship. **(B)** The density visualization map of co-authorship. **(C)** The citation network visualization map of cited authors. **(D)** The citation density visualization map of cited authors.

More than 40,000 researchers had participated in the study of artificial intelligence in sepsis. We used the VOSviewer to survey the network visualizations of authors and cited authors, as shown in Figures 4A, B. Table 2 showed that the top 10 authors and co-cited authors. Among them, the top three authors of scientific research paper production were Schuetz, Philipp (*n* = 35), Mueller, Beat (*n* = 28), Wong, Hector r (*n* = 27). The articles published by Vincent, Jl (*n* = 1498), Dellinger, Rp (*n* = 989), Bone, Rc (*n* = 910) were cited the most. Figures 4C, D showed the cluster density visualization map. Clusters with the same color represent similar research directions. In the author’s cluster density visualization map (Figure 4C), it was obvious that the red cluster Schuetz, Philipp occupied higher weight. In the visual map of cited author cluster density (Figure 4D), it was obvious that the red cluster Vincent and JL occupied a higher weight.

**TABLE 2:** The top 10 authors and cited authors with most publications on the application of artificial intelligence in sepsis from 2000 to 2021.

Rank	Author	Count	Total link strength	Rank	Cited author	Citations	Total link strength
1	Schuetz, Philipp	35	101	1	Vincent, Jl	1498	14381
2	Mueller, Beat	28	93	2	Dellinger, Rp	989	10123
3	Lorente, Leonardo	27	144	3	Bone, Rc	910	7152
4	Wong, Hector r.	27	192	4	Singer, M	906	7543
5	Jimenez, Alejandro	26	141	5	Knaus, Wa	789	6833
6	Kollef, Marin h.	26	43	6	Levy, Mm	729	7738
7	Weiss, Scott l.	25	167	7	Schuetz, P	573	7939
8	Christopher, Kenneth b.	22	38	8	Angus, Dc	570	6197
9	Martin, Maria m.	22	133	9	Stoll, Bj	437	1785
10	Sole-violan, Jordi	22	130	10	Kumar, A	434	5149

### 3.4. Analysis of journal and cited journal

**FIGURE 5:**
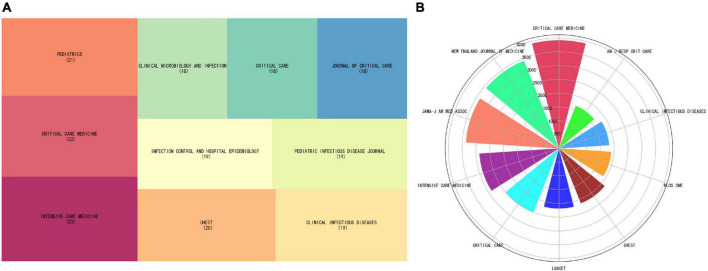
**(A)** The top 10 journals tree diagram. **(B)** The top 10 cited journals rose diagram.

More than a thousand academic journals had published articles related to the application of artificial intelligence in sepsis, among which *Intensive Care Medicine* (*n* = 23, IF 2021 = 41.787) ranked first, followed by *Critical Care Medicine* (*n* = 22, IF 2021 = 9.296), as shown in Table 3. Figure 5 visualizes the top 10 journals and cited journals by tree diagram and rose diagram, respectively. The JCR of the top 10 journals in terms of publication volume belongs to Q1/Q2, which provided high-quality and valuable literature in this field to a certain extent. The top three cited journals were *Critical Care Medicine* (*n* = 3918, IF 2021 = 9.296), *New England Journal of Medicine* (*n* = 3413, IF 2021 = 176.079), *Jama-Jam Med Assoc* (*n* = 3369, IF 2021 = 157.335). The top 10 journals and cited journals were all 80% from the USA, followed by 20% from the UK, as shown in Supplementary Figure 1.

**TABLE 3:** The top 10 journals and cited journals with most publications on the application of artificial intelligence in sepsis from 2000 to 2021.

Rank	Journal	Count	IF (2021)	JCR	Rank	Cited journal	Count	IF (2021)	JCR
1	Intensive Care Medicine	23	41.787	Q1	1	Critical Care Medicine	3918	9.296	Q1
2	Critical Care Medicine	22	9.296	Q1	2	New England Journal of Medicine	3413	176.079	Q1
3	Pediatrics	21	9.703	Q1	3	Jama-Jam Med Assoc	3369	157.335	Q1
4	Chest	20	10.262	Q1	4	Intensive Care Medicine	2881	41.787	Q1
5	Clinical Infectious Diseases	19	20.999	Q1	5	Critical Care	2503	19.334	Q1
6	Infection Control and Hospital Epidemiology	19	6.520	Q1	6	Lancet	2186	202.731	Q1
7	Pediatric Infectious Disease Journal	19	3.806	Q1	7	Chest	2149	10.262	Q1
8	Clinical Microbiology and Infection	18	13.310	Q1	8	Plos One	1896	3.752	Q2
9	Critical Care	18	19.334	Q1	9	Clinical Infectious Diseases	1822	20.999	Q1
10	Journal of Critical Care	18	4.298	Q2	10	Am J Resp Crit Care	1654	30.528	Q1

**FIGURE 6:**
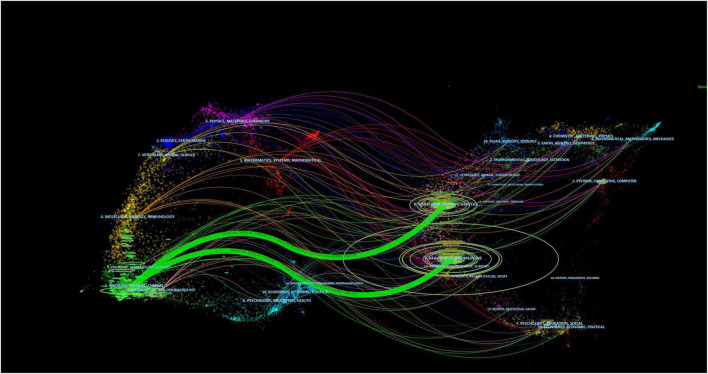
A dual-map overlap of journals on the application of artificial intelligence in sepsis.

The dual map overlay of the journal illustrated the topic distribution of the journal. The journals are on the left side of the map, while the cited journals are on the right. The colored paths indicate reference relevance, the flow and connection of knowledge from different research fields. We found two main citation paths. These two green path indicated that studies published in *Modular, Biology, Genetics and Health, Nursing, Medicine* were often cited in studies published in *Medicane, Medical, Clinical*, as shown in Figure 6.

### 3.5. Analysis of cited references

**FIGURE 7:**
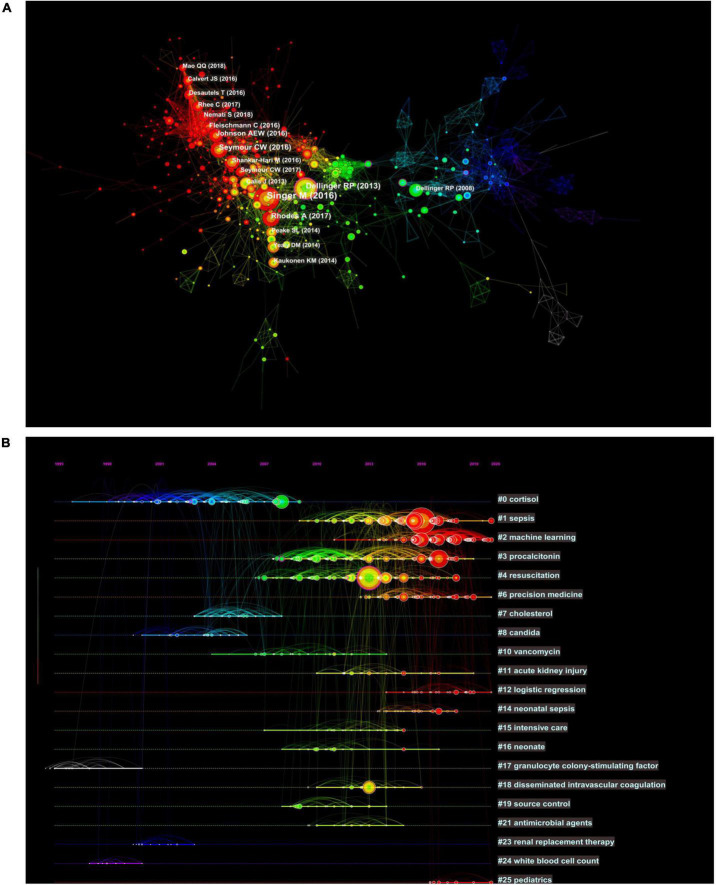
Co-citation network and timeline view of references cited by publications about artificial intelligence to sepsis. **(A)** References co-citation network. Circle node represents reference; the line between the nodes indicates the frequency of the two references being cited at the same time. **(B)** Timeline view of references. Each horizontal line represents a cluster; the circular nodes on the line represent the top three most cited references in this time slice. The timeline is shown at the top of the figure, and the year corresponding to the node is its publication time. Link between nodes represents the co-citation relationship.

**FIGURE 8:**
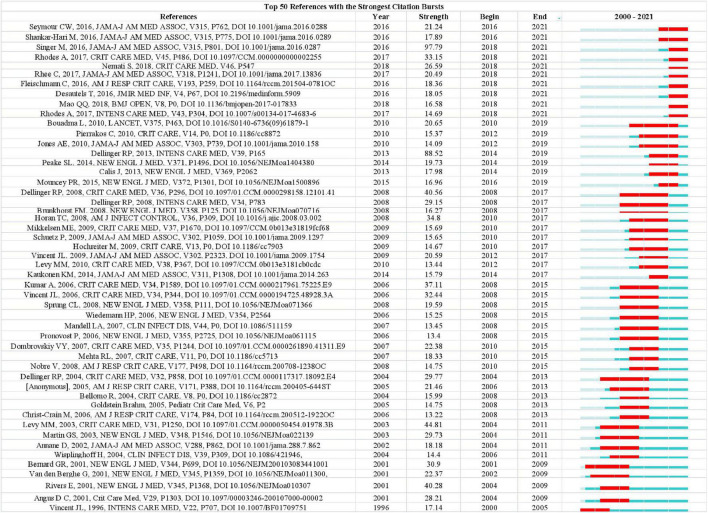
Ranking of references with the strongest citation bursts related to artificial intelligence in sepsis. The strength values reflect the frequency of citation. Red bars indicate a burst period for the references.

The analysis of the co-cited literatures reveals the authoritativeness of the research in this field and the great contribution of the authors. Figure 7A showed the visualization of the co-cited literatures, showing a total of 1,358 nodes and 4,834 links, with *Q*-value of 0.9069 and mean (Q, S) = 0.8598. As shown in Table 4, the characteristics of the top 10 highly cited literatures on the application of artificial intelligence in sepsis were summarized. The most co-cited literatures were published by Singer M et al. (*n* = 839), followed by Dellinger RP et al. (*n* = 336) and Rhodes A et al. (*n* = 228). The citations with higher centrality were published by Dellinger RP et al. (Centrality = 0.13), followed by the articles published by Singer M et al. (Centrality = 0.09). In addition, the co-cited literatures were divided into 25 clusters according to the index items, and the maximum 22 clusters were extracted using the log-likelihood ratio algorithm (LLR). Figure 7B showed their different timeline views, including Cortisol (cluster #0), Sepsis (cluster #1), Machine Learning (cluster #2), Procalcitonin (cluster #3), Resuscitation (cluster #4), Precision Medicine (cluster #6), Acute kidney injury (cluster #11), Logistic regression (cluster #12), etc., the mean silhouette value of each cluster was above 0.8, indicating that the cluster quality was credible and significant. Table 5 lists the details of the largest 22 clusters in the co-cited network, illustrating the temporal scientific relevance of the co-cited references. The strongest citation bursts analysis can find peer attention emerging concepts and future trends. A citation of literature bursts first appeared in 2000, the latest in 2018. The strongest burst (Strength: 97.79) was in the 2016 article. A total of 10 references had outbreaks that continued into 2021 (Figure 8).

**TABLE 4:** The top 10 cited references with most publications on the application of artificial intelligence in sepsis from 2000 to 2021.

Rank	Title	Author	Year	Journal	Count	Centrality
1	The third international consensus definitions for sepsis and septic shock (sepsis-3)	Singer et al.	2016	Jama-Jam Med Assoc	839	0.09
2	Surviving sepsis campaign: international guidelines for management of severe sepsis and septic shock, 2012	Dellinger et al.	2013	Intens Care Med	336	0.13
3	Surviving sepsis campaign: international guidelines for management of sepsis and septic shock: 2016	Rhodes et al.	2017	Crit Care Med	228	0.03
4	Assessment of clinical criteria for sepsis for the third international consensus definitions for sepsis and septic shock (sepsis-3)	Seymour et al.	2016	Ama-Jam Med Assoc	205	0.01
5	MIMIC-III, a freely accessible critical care database	Johnson et al.	2016	Sci Data	158	0.01
6	Assessment of global incidence and mortality of hospital-treated sepsis. Current estimates and limitations	Fleischmann et al.	2016	Am J Resp Crit Care	119	0
7	Developing a new definition and assessing new clinical criteria for septic shock: for the third international consensus definitions for sepsis and septic shock (sepsis-3)	Shankar-Hari et al.	2016	Jama-Jam Med Assoc	106	0
8	An Interpretable Machine Learning Model for Accurate Prediction of Sepsis in the ICU	Nemati et al.	2018	Crit Care Med	105	0.02
9	Surviving sepsis campaign: international guidelines for management of severe sepsis and septic shock: 2008	Dellinger et al.	2008	Crit Care Med	87	0.09
10	Incidence and trends of sepsis in US hospitals using clinical vs claims data, 2009–2014	Rhee et al.	2017	Jama-Jam Med Assoc	87	0.03

**TABLE 5:** Top 22 largest clusters of co-cited references on the application of artificial intelligence in sepsis from 2000 to 2021.

Cluster ID	Size	Silhouette	Mean (year)	Top terms (log-likelihood ration, *p*-level)
0	167	0.903	2002	Cortisol (32.26, 1.0E-4); machine learning (27.82, 1.0E-4); hypoglycemia (27.64, 1.0E-4); hyperglycemia (27.64, 1.0E-4); adrenal insufficiency (23.02, 1.0E-4)
1	119	0.806	2014	Sepsis (71.59, 1.0E-4); qsofa (39.44, 1.0E-4); emergency department (30.41, 1.0E-4); sirs (21.04, 1.0E-4); procalcitonin (19.08, 1.0E-4)
2	119	0.888	2017	Machine learning (145.13, 1.0E-4); artificial intelligence (40.39, 1.0E-4); deep learning (34.87, 1.0E-4); mortality (25.95, 1.0E-4); procalcitonin (24.41, 1.0E-4)
3	102	0.895	2012	Procalcitonin (208.77, 1.0E-4); antibiotic stewardship (66.41, 1.0E-4); biomarker (27.1, 1.0E-4); biomarkers (23.45, 1.0E-4); machine learning (20.21, 1.0E-4)
4	92	0.818	2012	Resuscitation (39.16, 1.0E-4); fluid therapy (24.39, 1.0E-4); machine learning (21.16, 1.0E-4); lactate (16.67, 1.0E-4); hydroxyethyl starch (12.68, 0.001)
6	61	0.899	2016	Precision medicine (28.73, 1.0E-4); personalized medicine (19.45, 1.0E-4); procalcitonin (16.64, 1.0E-4); endotype (14.79, 0.001); cluster analysis (12.45, 0.001)
7	41	0.959	2005	Cholesterol (16.94, 1.0E-4); prognosis (13.39, 0.001); natriuretic peptides (13.15, 0.001); b-type natriuretic peptide (13.15, 0.001); ventilator-associated pneumonia (10.3, 0.005)
8	38	0.981	2003	Candida (40.68, 1.0E-4); candida albicans (17.87, 1.0E-4); colonization (14.08, 0.001); very low birth weight (14.08, 0.001); extremely low birth weight (14.08, 0.001)
10	37	0.959	2009	Vancomycin (23.23, 1.0E-4); enterococcus faecium (23.23, 1.0E-4); mrsa (23.23, 1.0E-4); bacteremia (10.55, 0.005); clinical protocols (8.87, 0.005)
11	26	0.998	2013	Acute kidney injury (70.31, 1.0E-4); cirrhosis (20.2, 1.0E-4); dialysis (13.46, 0.001); nomogram (12.1, 0.001); diabetes (9.71, 0.005)
12	25	0.972	2017	Logistic regression (20.83, 1.0E-4); sepsis-induced coagulopathy (13.87, 0.001); coagulation (7.33, 0.01); mimic-iii (7.33, 0.01); sequential organ failure assessment (7.33, 0.01)
14	24	0.999	2015	Neonatal sepsis (42.28, 1.0E-4); early onset sepsis (24.62, 1.0E-4); neonatology (16.05, 1.0E-4); chorioamnionitis (16.05, 1.0E-4); risk factor (14.69, 0.001)
15	24	0.976	2012	Intensive care (18.95, 1.0E-4); malnutrition (16.23, 1.0E-4); uganda (16.23, 1.0E-4); mortality (14.24, 0.001); critical care (13.95, 0.001)
16	21	0.997	2010	Neonate (24.8, 1.0E-4); group b streptococcus (17.58, 1.0E-4); neonatal infection (16.14, 1.0E-4); recurrence (12.36, 0.001); intrapartum antibiotics (12.36, 0.001)
17	21	0.981	1997	Granulocyte colony-stimulating factor (10.39, 0.005); leukemia inhibitory factor (10.39, 0.005); cytokine (10.39, 0.005); granulocyte-macrophage colony-stimulating factor (10.39, 0.005); colony-stimulating factor (10.39, 0.005)
18	18	0.985	2013	Disseminated intravascular coagulation (39.14, 1.0E-4); thrombomodulin (17.82, 1.0E-4); atrial fibrillation (15.64, 1.0E-4); outcomes assessment (14.83, 0.001); amiodarone (14.83, 0.001)
19	17	0.994	2010	Candida (19.67, 1.0E-4); source control (16.42, 1.0E-4); antifungal therapy (16.42, 1.0E-4); candidiasis (10.94, 0.001); candidemia (9.79, 0.005)
21	17	0.969	2012	Antimicrobial agents (13.59, 0.001); bacteremia (11.23, 0.001); quality measurement (9.52, 0.005); extended-spectrum beta-lactamase (9.52, 0.005); acute care hospital (9.52, 0.005)
23	12	0.995	2000	Renal replacement therapy (18.26, 1.0E-4); acute renal failure (14.46, 0.001); serum albumin (10.98, 0.001); prognostic scores (10.98, 0.001); acute (10.98, 0.001)
24	11	0.997	1998	White blood cell count (22.27, 1.0E-4); multiple trauma (11.08, 0.001); inflammatory mediators (11.08, 0.001); multivariate analysis (11.08, 0.001); hypocalcemia (11.08, 0.001)
25	9	0.995	2017	Pediatrics (14.35, 0.001); klebsiella pneumoniae (13.59, 0.001); children (10.2, 0.005); infants (9.52, 0.005); ethiopia (9.52, 0.005)

### 3.6. Analysis of keywords

**FIGURE 9:**
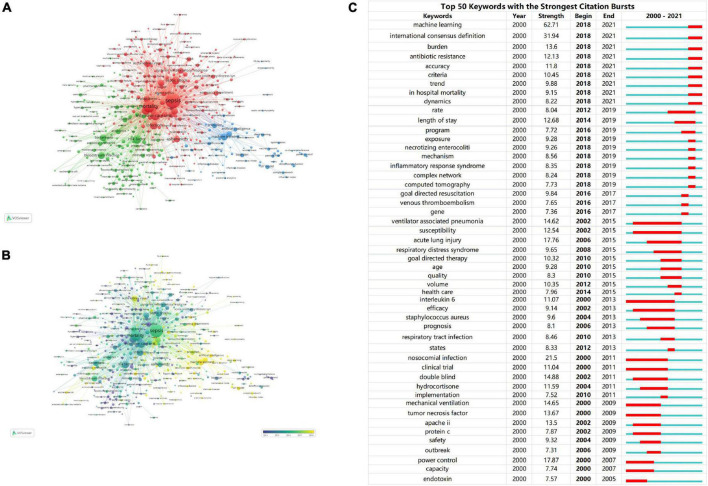
**(A)** The network visualization map of keywords. The 469 keywords were divided into three clusters. Each cluster represents a different research topic. **(B)** The overlay visualization map of keywords. The color ranges from purple to green to yellow, indicating the years of keyword appearance from early to late. **(C)** Ranking of keywords with the strongest citation bursts related to artificial intelligence in sepsis. The strength values reflect the frequency of citation. Red bars indicate a burst period for the references.

We analyzed more than 10,000 author keywords. Among them, 469 keywords had a frequency of 8 or more. Table 6 showed the top 10 keywords with the highest frequency. Those with frequencies above 200 were sepsis, mortality, intensive care unit, outcome, risk factor, septic shock, infection, machine learning, bacteremia, acute kidney injury respectively. The VOSviewer was used to build network visualizations and overlay visualizations, as shown in Figure 9. Each color of the network visualization graph represents a cluster, and nodes with common attributes are divided into a color-coded cluster. The 469 keywords were divided into three clusters, represented in green, blue, and red, as shown in Figure 9A. The red cluster mainly focused on the study of complications of sepsis, such as acute kidney injury, acute respiratory distress syndrome, acute renal failure, etc. The blue cluster mainly focused on the artificial intelligence technology, such as machine learning, bayesian, deep learning, neural network, random forest, etc. The green cluster mainly focused on the research of risk factors and treatment, such as age, hyperglycemia, HIV, obesity, beta-lactams, vancomycin, daptomycin, amikacin, linezolid, etc. Figure 9B was a superimposed visualization map. Different colors corresponded to the years of keyword appearance. The color ranges from purple to green to yellow, indicating the years of keyword appearance from early to late. In recent years, the keywords which appeared more frequently are machine learning, deep learning, antibiotic resistance, *Escherichia coli*, antibiotic stewardship, complex Network, early prediction. Supplementary Figure 2 visualized the top 10 keywords through the rose chart. CiteSpace was used to analyze keywords that strongly cite explosive growth, as shown in Figure 9C. The green line shows the time period between 2000 and 2021, and the red line shows the time period when the keywords burst. Keywords that surged in 2018–2019 included complex network (strength: 8.24) and inflammatory response syndrome (strength: 8.35). 2018–2021 showed more valuable keywords were machine learning (strength: 62.71), antibiotic resistance (strength: 12.13), accuracy (strength: 11.8).

**TABLE 6:** The top 10 keywords with most publications on the application of artificial intelligence in sepsis from 2000 to 2021.

Rank	Keywords	Count
1	Sepsis	1355
2	Mortality	792
3	Intensive care unit	338
4	Risk factor	319
5	Septic shock	308
6	Machine learning	231
7	Infection	218
8	Outcome	209
9	Prognosis	196
10	Acute kidney injury	188

## 4. Discussion

In this study, we retrieved publications on the application of artificial intelligence to sepsis from the WOSCC database over the past 22 (2000–2021) years. After excluding studies that did not meet the screening criteria, a total of 8,481 publications were included. We used Microsoft excel 2019, CiteSpace, VOSviewer, COOC software to analyze the annual number of publications, countries/regions, institutions, authors, journals, cited journals, cited references, and keywords to obtain an overview of research, development trends and future research hotspots in this field.

### 4.1. General information

From the results, it can be seen that the number of papers related to the application of artificial intelligence to sepsis is generally on the rise, in particular, the number of papers published in the past 5 years had accounted for half of the total number of publications, and the frequency of citations had exceeded 20,000 times after 2018. This showed that this topic had received a lot of attention in recent years.

According to the analysis of countries/regions and institutions, it can be seen that the USA, China, UK were the main research countries, among which the USA was the leading country in this field. Nine out of ten institutions were from the USA which had the most cooperation with other countries. For example, Harvard University from the USA had published the most articles and cooperated with other institutions the most. The cooperation between the agencies was relatively close. For developing countries, there was a large volume of publications, but its cooperation with other countries was not so close, such as China, suggesting that we should actively cooperate with other countries.

Each of the top 10 active authors published at least 22 publication. Professor Schuetz, Philipp from Switzerland ranked first, followed by Mueller, Beat, and Wong, Hector r from Switzerland and the USA, respectively. Schuetz, Philipp’s main research area was procalcitonin as a biomarker in the diagnosis and treatment of sepsis. Schuetz, Philipp et al. proposed specific procalcitonin algorithms for patients with low, moderate, and high sensitivity to reduce excessive antibiotic exposure in patients with respiratory infections and sepsis in 2011 (30). The Shapiro-Procalcitonin algorithm had been validated prospectively in 2020 to be highly valuable in predicting bacteremia, increasing the positive rate of true blood culture from 15 to 26% (31). Professor Vincent Jl ’s articles were the most cited among the top 10 cited authors, far more than other scholars. Vincent Jl had participated in the publication of high-level, high-quality consensus, clinical studies and reviews related to polysepsis, such as *The Third International Consensus Definitions for Sepsis and Septic Shock (Sepsis-3), Consensus on circulatory shock and hemodynamic monitoring*, *Vitamin C for Sepsis and Acute Respiratory Failure*, etc. (1, 32, 33). This showed that Vincent Jl had a deep research achievement in the field of sepsis.

*Intensive Care Medicine* magazine had the highest impact factor and the largest number of articles published in this study. This showed that *Intensive Care Medicine* was the top magazine in the field, offering high-quality articles. It may also attract more articles to the magazine in the future. In addition, *Critical Care Medicine* had been cited the most times, indicating that the journal still had a certain influence in this field. It was also worth noting that most JCR partitions, whether journal or cited journal, were Q1/Q2. These data will help researchers to provide choices when they submit articles about the application of artificial intelligence in sepsis in the future.

### 4.2. Knowledge base

It is pertinent to note that the number of citations reflects the amount of influence the relevant literature has on the topic. Using a combination of manual literature reading and information extraction, this study discovered that among the 10 literatures with the greatest citation rate, with the exception of one article published in 2018, a machine learning model was utilized to precisely predict sepsis in the critical care unit (34). Additionally, a guide to the MIMIC-III public database was released in 2016 (35). Other articles covered the recent definition of sepsis as well as related diagnostic and therapeutic introductions (1, 36–40). These articles indicated that sepsis was still being defined, diagnosed, and treated in an evolving manner.

As shown in the timeline view of references (Figure 7B), the two decades can be roughly divided into three phases. In the first stage, from 2000 to 2009, cortisol, cholesterol, candida, granulocyte colony-stimulating factor, renal replacement therapy, and white blood cell count were clustered as reference words. The focus of previous studies has been primarily on screening and identifying sepsis biomarkers. It has been demonstrated that granulocyte colony-stimulating factor, neutrophil CD64, interleukin-1 receptor antagonist, monocyte chemoattractant protein-1, procalcitonin, interleukin-6, lactic acid as well as other biomarkers provided a new data space for early prediction of sepsis using machine learning methods (41). In the second stage, which lasted from 2004 to 2014, the cluster words were vancomycin, intensive care, neonates, and acute kidney injury. The main focus of research in this decade was on drug treatment and complications associated with sepsis. Sepsis has always been a hot topic in the field of critical care medicine with respect to prevention, diagnosis, and treatment of AKI (42). There has been some success in identifying the subtypes of AKI associated with sepsis using machine learning (43–45). In spite of this, there remains a need for further research in order to determine the optimal treatment for different subtypes of AKI, as well as whether this treatment can improve the prognosis of patients. In the third stage, the period from 2010 to 2021 was considered. As the reference cluster words were sepsis, machine learning, procalcitonin, resuscitation, and precision medicine, it indicated that in recent years, the treatment of sepsis has moved toward precision treatment (46). The main treatment for sepsis consists of fluid resuscitation, anti-infective therapy, improvement of oxygen delivery and protection of organ function (39). Although clinical guidelines are used to guide treatment, the heterogeneity of sepsis makes clinical efficacy insignificant (47). Among these factors, clinical complexity, subjective differences in examination indicators, physician experience, and patient status all influenced the treatment plan for sepsis. These variables are unaffected by machine learning, which is capable of providing precision therapy (48, 49). In situations when the ideal length of treatment is unclear, biomarkers like procalcitonin are advised in addition to clinical examination to help decide when to stop using antibiotics (50).

As shown in the keyword cluster analysis in Figure 9A, Vosviewer divides keywords into three clusters, including “risk factors” in addition to the subject words “machine learning” and “sepsis.” Moon Seong Baek et al. analyzed through machine learning that relatively older age and lower body temperature were risk factors for death in sepsis patients (51). Traditional machine learning algorithms such as Neural network, Bayes and Random forest had been used in early diagnosis, precise treatment and prognosis assessment of sepsis, these training models had shown excellent performance (52–54). However, compared with traditional machine learning algorithms, deep learning had been widely loved by researchers in recent years due to its strong learning ability and ability to deal with complex problems. Simon Meyer Lauritsen et al. developed an early deep learning algorithm for identifying sepsis that can learn key factors and interacting features from the raw event sequence data itself without relying on labor-intensive feature extraction efforts (55). Zhongheng Zhang et al. identified two types of sepsis based on deep learning cluster analysis. The first type was characterized by immunosuppression with high mortality, the second group, which is relatively immunocompetent, also showed different mortality outcomes and responses to hydrocortisone treatment (56). Therefore, the in-depth development of machine learning algorithm provides powerful technical support for the diagnosis and treatment of sepsis.

### 4.3. Research hotspots and trends

The dynamic nature of trends in this field are partially characteristic of the references with citation bursts. Statistics from CiteSpace found that there were 10 articles that broke out in recent years (2018–2021), which can be roughly divided into two parts of research content. The first part covers the definition, diagnosis, epidemiology, and management of sepsis. The representative reference with the strongest currently ongoing citation bursts was an article published by Singer M et al. (1), the definition of sepsis according to version 3.0 of this article is a condition of the body’s reaction to infection that can result in life-threatening organ malfunction. Sepsis 1.0 and 2.0 were defined as SIRS caused by infection, which was characterized by excessive emphasis on infection, while sepsis 3.0 was more closely to the nature of sepsis, which focused on the dysregulation of the body’s response to infection and organ dysfunction (1). The definition of septic shock had also been redefined: sepsis occurs with severe circulatory, cellular, and metabolic abnormalities sufficient to significantly increase the case fatality rate, recognizing the importance of the cellular abnormalities compared to the previous definition (36). Seymour, Christopher W et al. evaluated the newly defined clinical standard performance of sepsis, because there was no gold standard for sepsis detection, the use of SIRS, SOFA, LODS, qSOFA to evaluate potential clinical criteria. The results showed that the predictive validity of SOFA in-hospital mortality was not significantly different from the more complex LODS in ICU patients with suspected infection, but was statistically greater than SIRS and qSOFA. Therefore, SOFA can be used as a prompt to consider sepsis (37). *Critical Care Medicine* and *Intensive Care Medicine* jointly published online the *Surviving Sepsis Campaign: International Guidelines for Management of Sepsis and Septic Shock: 2016*, which provides 93 statements on early management and resuscitation of sepsis, of which 32 are strong recommendations (39, 57). The second part deals with emerging technologies for predicting sepsis. Shamim Nemati et al. developed and verified that the AISE algorithm can use real-time data (65 indicators) from the ICU to predict the onset of sepsis 4–6 h in advance, and can take effective measures in a timely manner based on the prediction results (34). Desautels et al. proposed the InSight algorithm in 2016, using 8 clinical vital signs (systolic blood pressure, pulse pressure, heart rate, respiration rate, temperature, SpO2, age, and GCS) predicts sepsis patients in the ICU, showing that InSight outperforms qSOFA, SIRS, and MEWS scores (58). Similarly, Qingqing Mao et al. also used a machine learning-based algorithm InSight using only six clinical vital sign measures (systolic blood pressure, diastolic blood pressure, heart rate, respiratory rate, SpO2 temperature) can identify and predict the occurrence of sepsis, providing the basis for improving patient outcomes (59). Citation burst analysis shows that defining sepsis is challenging, and there is currently no single standard to identify sepsis, which may need to be explored with the help of emerging research tools, and it is believed that the in-depth development of artificial intelligence algorithms in the future will inject new life into the field.

Logistic Organ Dysfunction System (LODS); Sequential (Sepsis-related) Organ Failure Assessment (SOFA); Systemic inflammatory response syndrome (SIRS); Quick Sequential (Sepsis-related) Organ Failure Assessment (qSOFA); Artificial Intelligence Sepsis Expert (AISE); Glasgow Coma Score (GCS); Peripheral capillary oxygen saturation (SpO2); Acute kidney injury (AKI).

Keywords are an important part of an article and the embodiment of the core idea of an article (60).

CiteSpace is used for burst detection of keywords. We identified machine learning, antibiotic resistance, accuracy that were meaningful for this field.

#### 4.3.1. Machine learning

Machine learning develops tools for early diagnosis, precision treatment, and prognostic assessment of sepsis by mining clinical data such as demographics, laboratory indicators, comorbidities, microbial culture results, gene. Shamim Nemati et al. developed an early prediction algorithm for sepsis, which can use EMR data combined with high-resolution time series dynamics of heart rate and blood pressure to predict sepsis 4 h in advance (34). Nianzong Houden et al. used XGboost to construct a death model for predicting sepsis patients. The results showed that the predictive performance of XGboost model was better than that of traditional logistic regression model and SAPS-II scoring model (61). Of course, machine learning was not limited to obtaining data from EHR to predict the occurrence of sepsis, but also to predict the occurrence of sepsis from the RNA level. James Ducharme et al. proposed a blood-based 29-host mRNA test that combines machine learning with a rapid care point platform in less than 30 min to quickly diagnose acute infections and sepsis and predict the severity of the disease (62). In the published research, the model characteristics of machine learning prediction were good, but if it was effectively converted into clinical application, it was a question worthy of our consideration. Jennifer C. Ginestra’s team used machine learning to develop the first predictive tool (EWS 2.0) for evaluating non-ICU sepsis patients and deployed it in hospitals for prospective validation (63). Although EWS 2.0 had excellent predictive power for severe sepsis and septic shock, the results of clinical application were not satisfactory (56). The authors summarized the current problems of EWS2.0 from four aspects: patients, clinicians, machine learning algorithms, and alert response (63). Maybe we can also learn from it. Coincidentally, Kollef et al. deployed powerful real-time early warning scores in hospitals and found that alerts transmitted in the intervention group did not reduce either the ICU transfer rate or the mortality rate (64). But there was a good news from a recent study, in a prospective study, patients who received a Targeted Real-time Early Warning System (TREWS) within 3 h had reduced mortality, organ failure, and length of hospital stay compared with patients who did not receive a warning at 3 h (65). Machine learning techniques have good sensitivity and specificity, and may still be the dominant technology in the future to provide supporting information for clinical decision-making.

#### 4.3.2. Antibiotic resistance

For patients with sepsis, it is beneficial to start antibiotic treatment as early as possible, but inadequate and unnecessary empirical broad-spectrum antibiotic treatment may lead to antibiotic resistance and increase the risk of death (66). A retrospective cohort study had shown that multidrug resistance was an important determinant of non-initial appropriate antibiotic treatment and was associated with a threefold increase in the risk of hospital death (67). Therefore, we need to identify patients with drug-resistant pathogens early and accurately use broad-spectrum antibiotics for empirical sepsis treatment (68). Mathew Stracy et al. combined the results of bacterial genome sequencing with clinical data to develop a machine learning algorithm for personalized antibiotic recommendation to predict whether patients will develop drug resistance (69). Ohad Lewin-Epstein et al. used machine learning methods to predict the resistance of ceftazidime, gentamicin, imipenem, ofloxacin, and sulfonamides based on antibiotic resistance results of bacterial culture and EMR data (70). More importantly, the model of antibiotic resistance can be successfully applied in clinical practice. A prospective study by Marion Elligsen et al. showed that the individualized prediction model based on drug resistance will affect the antibiotic selection of G-bacteria and effectively carry out antibiotic downgrade treatment (71). This was a successful study, but there are few reports on such studies. In the future, it may be necessary to develop more, more accurate and more personalized antibiotic resistance models and apply them effectively in clinical practice.

#### 4.3.3. Accuracy

The diagnosis and treatment of sepsis has entered the era of precision, and the continuous updating of the definition of sepsis is the basis for individualized treatment of sepsis. With the development of big data and artificial intelligence technology, it has provided strong support for the diagnosis and treatment of sepsis. In the early stage of sepsis, artificial intelligence technology can accurately, quickly and timely predict the occurrence of sepsis, providing enough time for the treatment of sepsi (34). Due to the high heterogeneity of sepsis, its clinical treatment was controversial. Christopher W. et al. included the data of 63,858 patients and deduced and verified four types of sepsis phenotypes (α, β, γ, and δ), which had their own characteristics, type α is characterized by the lowest amount of vasopressor medication; type β is characterized by older age, chronic diseases and renal insufficiency; type γ is characterized by pronounced manifestations of inflammation, pulmonary dysfunction; type δ is characterized by liver dysfunction and septic shock (72). It provides accurate treatment direction for sepsis patients with different phenotypes. The benefit of corticosteroids in the treatment of septic patients is controversial. Romain Pirrachio et al. used machine learning methods to conduct personalized evaluation of treatment effect to determine which patients were suitable for corticosteroid treatment (73). This greatly improves the therapeutic effect.

At present, precision diagnosis and treatment has also become a frontier topic in the field of sepsis. Machine learning can use its strengths to develop personalized sepsis diagnosis and treatment tools to help clinicians make better decisions. However, there are also challenges, such as poor or missing clinical data recording, retrospective data used in most studies, small sample sizes, false positive results, absence of standard for unifying evaluation model, lack of innovative model, missing external validation of model, and poor transparency and interpretability of model. In future work, we need to standardize clinical data recording, develop new algorithms, establish accurate, dynamic, real-time and practical models, and conduct large-scale prospective experiments to promote the clinical application of machine learning models.

## 5. Strengths and limitations

To our knowledge, this study was the first to systematically analyze the publications on the application of artificial intelligence in sepsis in an objective way. Various bibliometric software was used to analyze the research overview, research topics and research hotspots from multiple dimensions. Specifically, for developing countries, there was a large volume of publications, but its cooperation with other countries was not so close, this will help researchers in the field to realize the need for greater international and regional cooperation. At the same time, the analysis of cited references and keywords will help researchers understand popular research in the field, as well as identify potential research hotspots and future directions. Inevitably, this study has some limitations. First of all, the research publications in this paper only come from WOS, without considering CNKI and other databases. Secondly, the publication language type of this paper is limited to English, and important publications in other languages may be ignored. Last but not least, it may not have been enough time for some articles to be read and cited by interested authors when the comprehensive search was performed. Future research could better incorporate these factors. However, this study aimed to conduct a high-quality bibliometric analysis of artificial intelligence in sepsis, consequently, these limitations are unlikely to affect the present findings, which capture research trends in the field of interest.

## 6. Conclusion

This study conducted a comprehensive and objective analysis of the publications on the application of artificial intelligence in sepsis. Over the past 22 years, the annual number of publications had gradually increased exponentially, suggesting that research in this area remains a hot topic in the future. The USA was the most productive country, followed by China. However, greater cooperation is needed between countries, especially developing countries. In addition, it can be predicted that precise treatment of sepsis through machine learning technology is still research hotspot in this field.

## Data availability statement

The original contributions presented in this study are included in the article/Supplementary material, further inquiries can be directed to the corresponding authors.

## Author contributions

MT, FM, and J-YZ designed the study. J-YZ, CC, and K-XS collected and verified the data. CC, RL, and MT performed the software analysis. MT and FM wrote the first manuscript. YG and J-WW revised and approved the final version of the manuscript. All authors approved the final manuscript.
