# Supplementary material for: Research frontiers and trends in the application of artificial intelligence to sepsis: A bibliometric analysis
Source: Front Med (Lausanne). 2023 Jan 12;9:1043589. doi: 10.3389/fmed.2022.1043589 (PMC9878129; doi:10.3389/fmed.2022.1043589)
Supplement: Supplementary file 1 [file Data_Sheet_1.pdf]

1. The complete search strategy in WOSCC

TS=(sepsis OR “septic shock” OR ”systemic inflammatory response syndrome” OR SIRS OR “severe sepsis” OR “multiple organ dysfunction syndrome” OR “MODS” OR “Bloodstream Infection*” OR pyemia* OR pyohemia*OR “blood poisoning”OR“pyaemia*”OR“septicemia*”) AND TS=(algorithm* OR “decision trees”OR “decision support systems, clinical” OR “decision making,computer” OR “decision support techniques” OR “machine learning” OR “prediction model” OR “neural network” OR “deep learning” OR “artificial intelligence” OR AI OR “machine intelligence” OR bayesian OR “random forest” OR “support vector machine” OR SVM OR Xgboost OR adaboost OR “gradient boosting machine” OR “regression tree” OR “stepwise regression” OR “computer prediction” OR “prediction algorithm” OR “supervised learning” OR “unsupervised learning” OR “big data” OR “natural language” OR “logistic regression” OR “convolutional neural network” OR “ensemble learning” OR “reinforcement learning”)

2. The Complete data cleansing

In the country analysis: Wales, Scotland, North Ireland, and England were classified as the UK, and Taiwan was classified as Peoples R China.
In terms of institutions: for the organization of writing unification, such as, Univ Texas Southwestern med CTR Dalla was classified as Univ Texas SW med CTR Dallas; univ washington was classified as washington univ; univ paris 05 univ paris was classified as univ paris; univ paris 06 was classified as univ paris;univ paris 07 was classified as univ paris; cnr was classified as cnrs; peking union med coll was classified as peking union med coll hosp; natl sun yat sen univ was classified as sun yat sen univ;
In keywords analysis: synonymous keywords were merged, such as intensive care unit (ICU) was classified intensive care unit, acute kidney injury (AKI) was classified acute kidney injury;anti-bacterial agents was classified as antibiotics; antibacterial agents was classified as antibiotics; acute respiratory distress syndrome (ards) was classified as acute respiratory distress syndrome; antibiotic treatment was classified as antibiotic therapy; antimicrobial resistance was classified as antibiotic resistance; antimicrobial stewardship was classified as antibiotic stewardship ;antimicrobial therapy was classified as antibiotic therapy; artificial neural networks was classified as artificial neural network; bacteraemia was classified as bacteremia; bacterial infections was classified as bacterial infection; bayesian analysis was classified as bayesian; bayesian estimation was classified as bayesian; bayesian statistics was classified as bayesian; bayesian inference was classified as bayesian;biomarkers was classified as biomarker; blood cultures was classified as blood culture;bloodstream infections was classified as bloodstream infection; burns was classified as burn; candidemia was classified as candidaemia; case-control study was classified as case-control; clinical decision support systems was classified as clinical decision support system;clinical outcomeswas classified as clinical outcome;clinical trials was classified as clinical trial; cohort studies was classified as cohort study;comorbiditieswas classified as comorbidity; complex networks was classified as complex network;complications was classified as comorbidity; covid-19 pandemicwas classified as covid-19;costs was classified as cost;critically ill was classified as critical illness;cytokines was classified as cytokine; deep venous thrombosis was classified as deep vein thrombosis;diagnostics was classified as diagnosis;electronic health record was classified as electronic health records;electronic medical records was classified as electronic health records; endotoxemia was classified as endotoxin;enterococcus faecalis was classified as enterococcus faecium;epidemic models

was classified as epidemic model; extracorporeal membrane oxygenation (ecmo)was classified as extracorporeal membrane oxygenation;genetic algorithms was classified as genetic algorithm;glucocorticoids was classified as glucocorticoid;gram-negative bacilli was classified as gram-negative bacteria; guidelines was classified as guideline; healthcare-associated infections was classified as healthcare-associated infection;infants was classified as infant;intensive care unit (icu) was classified as intensive care unit;intra-abdominal infections was classified as intra-abdominal infection ;multidrug resistant was classified asmultidrug resistance;multidrug-resistantwas classified as multidrug resistance; multiple organ dysfunction syndrome (mods) was classified as multiple organ dysfunction syndrome;vasopressors was classified as vasopressor;urinary tract infections was classified as urinary tract infection;systemic inflammatory response syndrome (sirs) was classified as systemic inflammatory response syndrome;support vector machineswas classified as support vector machine;sequential organ failure assessment sequential organ failure assessment score;sepsis-3 was classified assepsis;septicemia was classified as sepsis;;risk factors was classified as risk factor ;readmissions was classified as readmission;prognostic factors was classified as prognostic factor;procalcitonin (pct)was classified as procalcitonin;preterm infants was classified as preterm infant;premature infants was classified as premature infant;Predictors was classified as predictor;predictive models was classified as predictive model;postoperative complications was classified as postoperative complication;particle filters was classified as particle filter;nosocomial infections was classified as nosocomial infection;neural networks was classified as neural network neonatal outcomes was classified as neonatal outcome;neonatal intensive care units was classified asneonatal intensive care unit;neonatal infections was classified as neonatal infection;aki was classified as acute kidney injury;Inpatients was classified as inpatient;infections was classified as infection;algorithm was classified as algorithms;sirs was classified as systemic inflammatory response syndrome;pediatric intensive care units was classified as pediatric intensive care unit;pediatric intensive care was classified as pediatric intensive care unit;logistic regression analysis was classified as logistic regression;intensive care units was classified as intensive care unit;outcomes was classified as outcome

3. Supplementary Figures

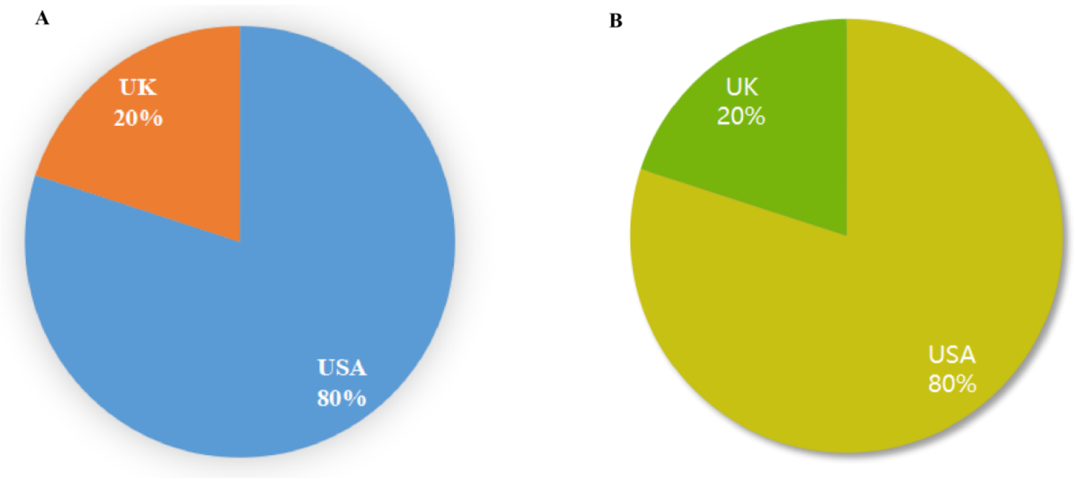

Figure S1. Percentage of countries that publish top 10 journals and cited journals

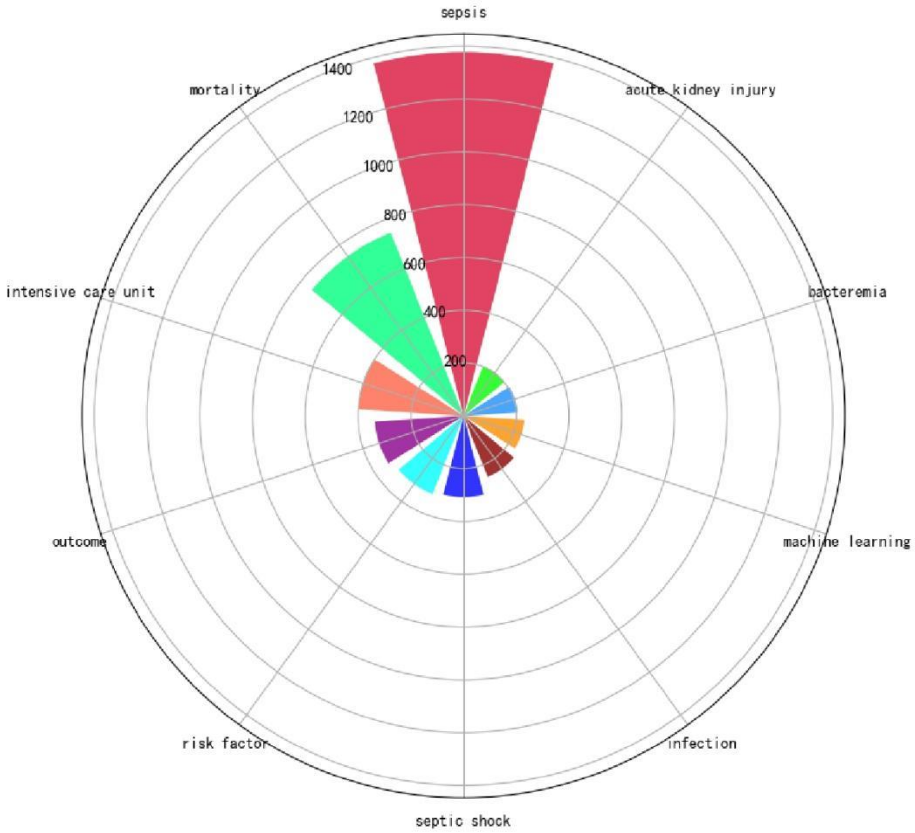

Figure S2 The top 10 keywords rose diagram
